# Supplementary material for: Comprehensive Physiology, Cytology, and Transcriptomics Studies Reveal the Regulatory Mechanisms Behind the High Calyx Abscission Rate in the Bud Variety of Korla Pear (Pyrus sinkiangensis ‘Xinnonglinxiang’)
Source: Plants (Basel). 2024 Dec 15;13(24):3504. doi: 10.3390/plants13243504 (PMC11677287; doi:10.3390/plants13243504)
Supplement: Supplementary file 1 [file plants-13-03504-s001.zip › Supplementary Materials(Figure).pdf]

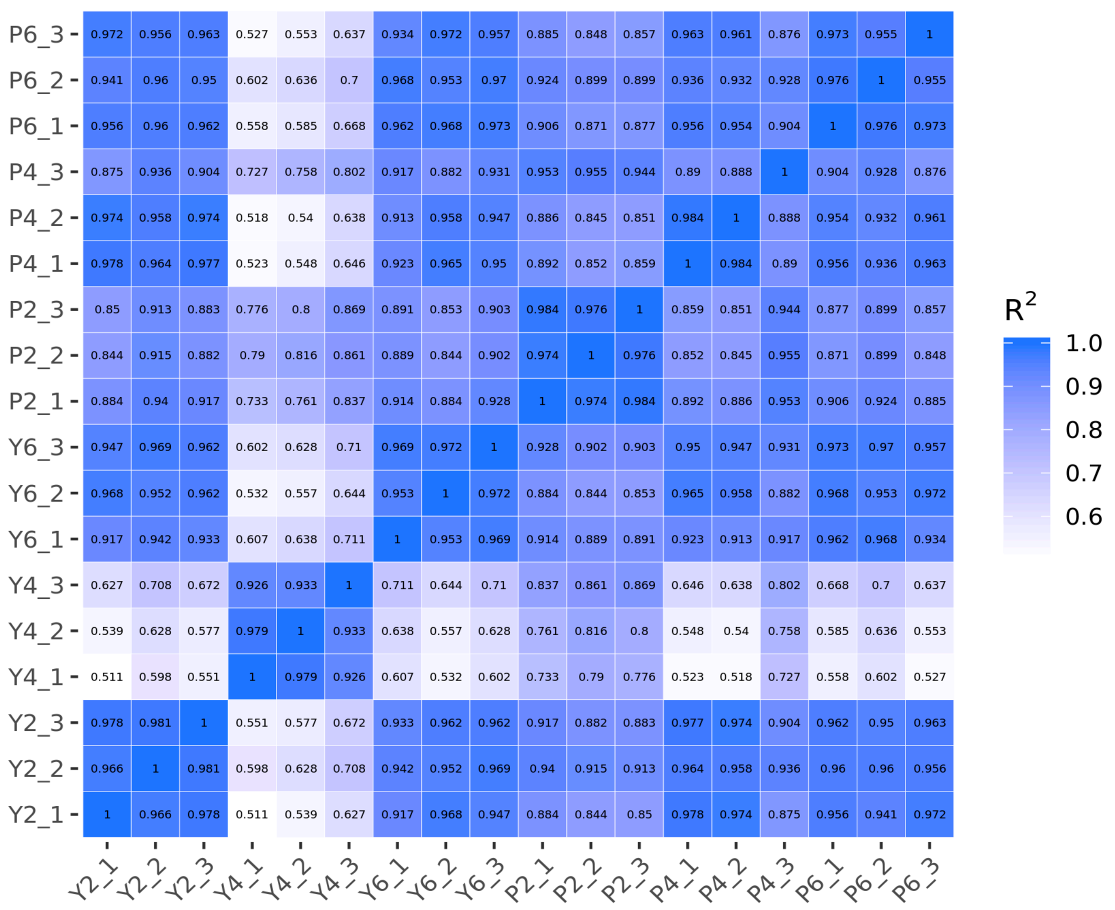

Figure S1. Correlation analysis between samples. Color from white to blue indicates the size of the correlation value. The closer the color is to white, the smaller the correlation value is. The closer the color is to blue, the greater the correlation value.

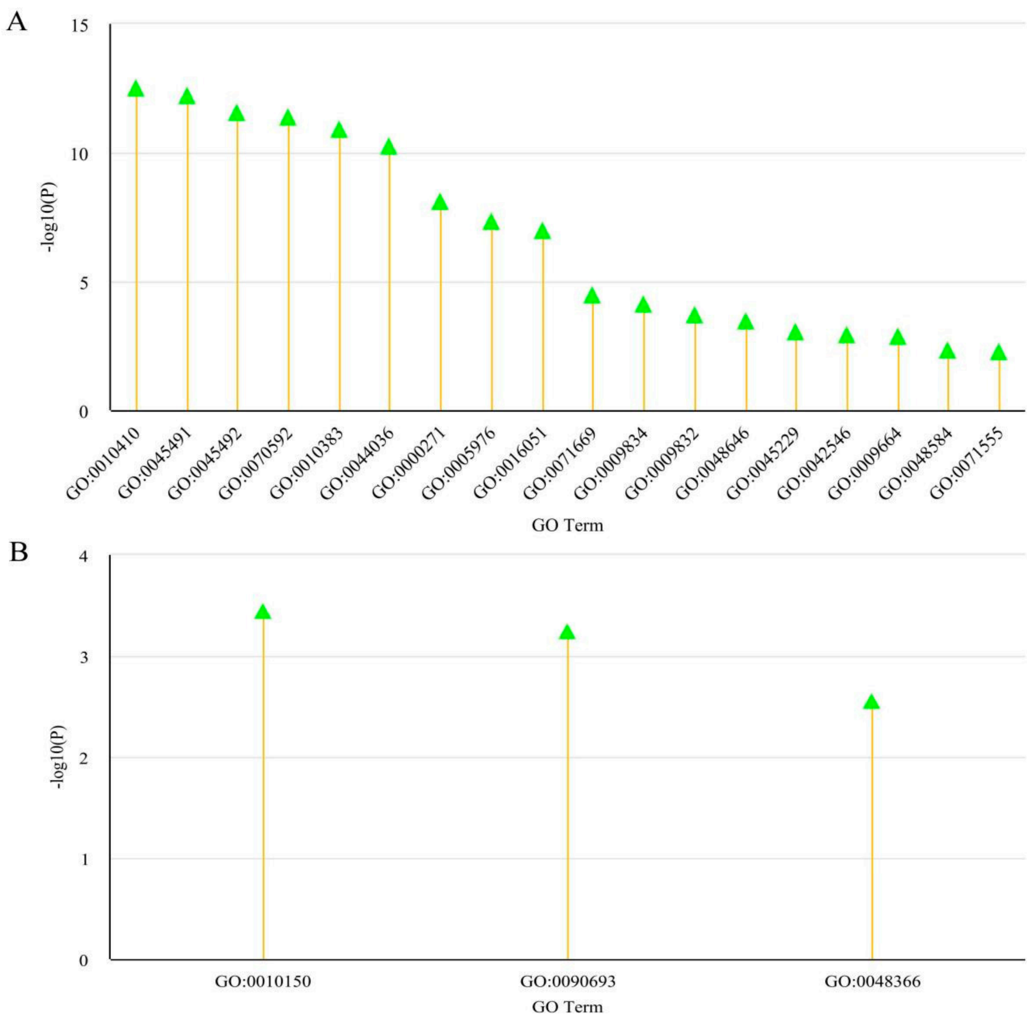

Figure S2. Core gene GO enrichment analysis of lollipops in key modules. A is the core gene GO enrichment result in the blue module, and B is the core gene GO enrichment result in the yellow module. The abscissa is GO Term and the ordinate is -log10(P) value, indicating the significance of enrichment. GO:0010410 is hemicellulose metabolic process; GO:0045491 is xylan metabolic process; GO:0045492 is xylan biosynthetic process; GO:0070592 is cell wall polysaccharide biosynthetic process; GO:0010383 is cell wall polysaccharide metabolic process; GO:0044036 is cell wall macromolecule metabolic process; GO:0000271 is polysaccharide biosynthetic process; GO:0005976 is polysaccharide metabolic process; GO:0016051 is carbohydrate biosynthetic process; GO:0071669 is plant-type cell wall organization or biogenesis; GO:0009834 is plant-type secondary cell wall biogenesis; GO:0009832 is plant-type cell wall biogenesis; GO:0042546 is cell wall biogenesis; GO:0048646 is anatomical structure formation involved in morphogenesis; GO:0045229 is external encapsulating structure organization; GO:0009664 is plant-type cell wall organization; GO:0071555 is cell wall organization; GO:0048584 is positive regulation of response to stimulus; GO:0010150 is leaf senescence; GO:0090693 is plant organ senescence; GO:0048366 is leaf development.

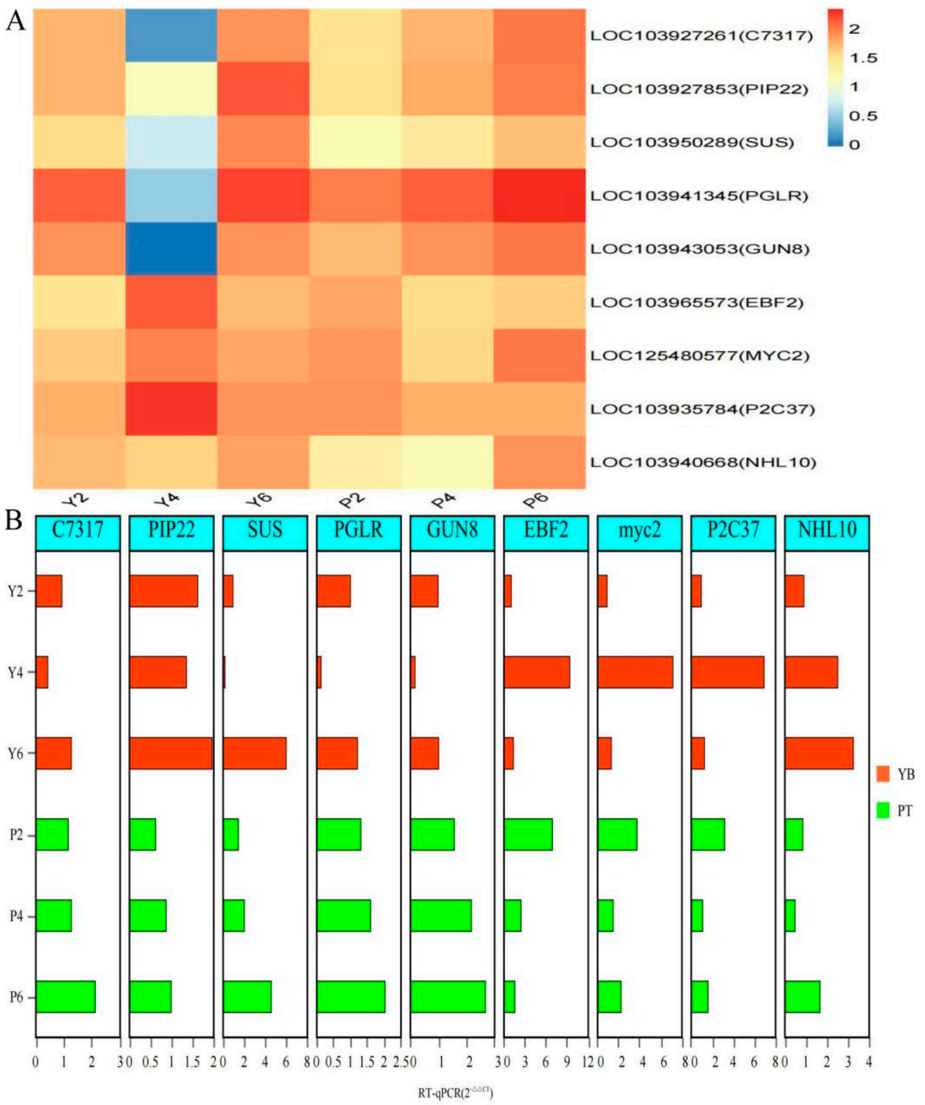

Figure S3. Real-time fluorescence quantitative verification of 19 genes. A is the transcriptome expression pattern heat map of 9 genes. The closer the color is to red, the higher the expression level is. B is the bar chart of real-time fluorescence quantitative expression results of 9 genes, the abscissa is the expression level, expressed as $2^{-\triangle\triangle Ct}$, and the ordinate is the sample name.

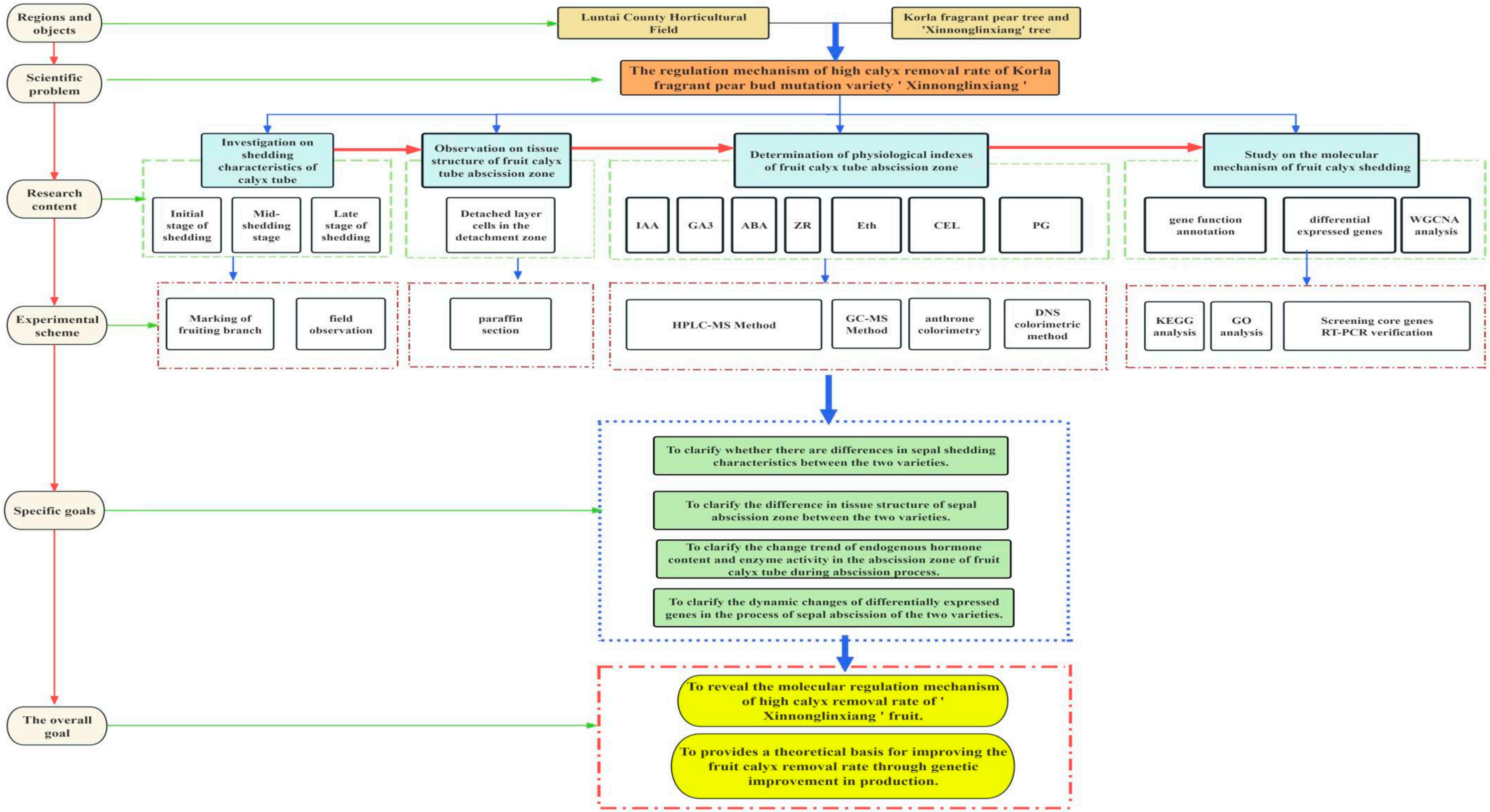

Figure S4. Experimental process and framework logic diagram.
