# Supplementary material for: Comprehensive Physiology, Cytology, and Transcriptomics Studies Reveal the Regulatory Mechanisms Behind the High Calyx Abscission Rate in the Bud Variety of Korla Pear (Pyrus sinkiangensis ‘Xinnonglinxiang’)
Source: Plants (Basel). 2024 Dec 15;13(24):3504. doi: 10.3390/plants13243504 (PMC11677287; doi:10.3390/plants13243504)
Supplement: Supplementary file 1 [file plants-13-03504-s001.zip › Supplementary Materials(Table).pdf]

Table S1. Transcriptome data statistics.

sample	clean reads	GC content(%)	Q30(%)	Total map(%)	Unique map(%)	Multi map(%)
P2_1	43936996	46.57	92.33	92.53%	90.29%	2.24%
P2_2	42815072	46.56	92.58	91.73%	89.64%	2.09%
P2_3	43218482	46.34	92.17	92.46%	90.29%	2.17%
P4_1	42315590	46.47	92.69	93.41%	91.23%	2.18%
P4_2	42622350	46.01	91.93	92.68%	90.54%	2.14%
P4_3	39235946	45.92	92.73	92.33%	90.21%	2.12%
P6_1	43308944	47.09	92.68	91.97%	88.76%	3.21%
P6_2	43902710	46.93	93.22	92.09%	89.72%	2.38%
P6_3	43868526	46.84	92.58	92.57%	90.23%	2.34%
Y2_1	40365188	46.90	92.60	92.83%	90.52%	2.31%
Y2_2	40870890	47.01	92.19	91.83%	89.75%	2.08%
Y2_3	42430946	47.19	92.53	92.54%	90.38%	2.16%
Y4_1	44228096	46.34	92.73	86.88%	84.93%	1.95%
Y4_2	41586208	46.68	92.46	90.03%	87.94%	2.09%
Y4_3	39960566	46.06	93.34	92.00%	89.90%	2.10%
Y6_1	42109766	46.77	92.68	90.97%	88.55%	2.43%
Y6_2	40751216	46.72	92.69	92.63%	90.05%	2.58%
Y6_3	42869142	46.63	91.97	91.86%	89.52%	2.34%

Table S2. GO enrichment analysis of DEGs in comparison group.

Comparison group	Category	GO ID	Description	Gene Ratio	Bg Ratio	Pvalue
YB2vsPT2	MF	GO:0016759	cellulose synthase activity	25/6934	37/15801	0.0030
	MF	GO:0004650	polygalacturonase activity	23/6934	48/15801	0.3362
YB4vsPT4	MF	GO:0016759	cellulose synthase activity	29/8558	36/15762	0.0009
	MF	GO:0004650	polygalacturonase activity	28/8558	52/15762	0.5823
YB6vsPT6	MF	GO:0016759	cellulose synthase activity	10/1485	37/15861	0.0016
	MF	GO:0004650	polygalacturonase activity	9/1485	48/15861	0.0320

Table S3. Functional annotation of 20 key core genes in the module.

Module	Key core genes	Gene description
Module blue	*C7317*（LOC103927261）	Cytochrome P450
	PIP22（LOC103927853）	aquaporin PIP2-2
	SAUR50（LOC103927977）	auxin-responsive protein SAUR50
	EIX2（LOC103945015）	receptor-like protein EIX1
	SUS（LOC103950289）	sucrose synthase
	ALE2（LOC103951862）	Receptor-like serine/threonine-protein kinase ALE2
	MA658（LOC125471232）	microtubule-associated protein
	PER64（LOC125473181）	peroxidase
	IRX10（LOC103950395）	beta-xylosyltransferase IRX10
	IRX9（LOC103934527）	beta-xylosyltransferase IRX9
	ESK1（LOC103936912）	xylan acetyltransferase
	GXM1（LOC103945016）	glucuronoxylan methyltransferase1
	GXM3（LOC103967497）	glucuronoxylan methyltransferase 3
Module blue	*M3K20*（LOC103935136）	mitogen-activated protein kinase kinase kinase
	FB316（LOC103964350）	F-box protein
	OXI1（LOC103932441）	serine/threonine-protein kinase
	ATL2（LOC103943691）	RING-H2 finger protein ATL2
	DURF2（LOC103954275）	E3 ubiquitin-protein ligase
	NHL10（LOC103940668）	NDR1/HIN1-like protein
	PUB21（LOC103967423）	U-box domain-containing protein

Table S4. RT-qPCR primer sequence information.

Gene ID	Forward primer	Reverse primer
TBA	GACCAGTGTTGCTGAGGTGT	ACTCTGCGCCCACTTCTTCATA
C7317	AACTCCAATAGCCACAAACCCA	TTGAAGCGAGCGATGAGGG
PIP22	GGAGCTGAGCACGGTGAGTTT	CTGGGATTTGTAGCCAATGACG
SUS	GTTGGCGGTAATTCTCCGTA	GGCAAGAAGCTGATGGGGTT
NHL10	ATGTGATTCAAGGCTCAAAGGTG	AGTCTGGAAAGTGCCAACTGAAT
EBF2	TTTCTCCGCAGGTAAAGACGAT	TCTTGCGAGGAGGAAATAGGAC
MYC2	AGTGAAGGACCCGGTGAATG	TCCGGTCATATCCGTTGTAGTC
P2C37	TCTTATCCTTGCCAGCGACG	ACCGAAATGAAACCACCTCGT
PGLR	GAGTCCTATGAAAACCCAAGTGAT	CCTTTGCCCTTCTGCTCTCA
GUN8	GCAGCCCTCTTCCTCCTTTCT	CCAGCAACGTCGTCGTGAAT
